# Increased Risk of Chronic Obstructive Pulmonary Disease in Patients with Hyperlipidemia: A Nationwide Population-Based Cohort Study

**DOI:** 10.3390/ijerph191912331

**Published:** 2022-09-28

**Authors:** Hao-Yu Yang, Li-Yu Hu, Hon-Jhe Chen, Ru-Yih Chen, Chang-Kuo Hu, Cheng-Che Shen

**Affiliations:** 1 Department of Family Medicine, Kaohsiung Veterans General Hospital, Kaohsiung 813414, Taiwan; 2 Department of Psychiatry, Taipei Veterans General Hospital, Taipei 112, Taiwan; 3 School of Medicine, National Yang Ming Chiao Tung University, Taipei 112, Taiwan; 4 Division of Neurosurgery, Department of Surgery, Chiayi Branch, Taichung Veterans General Hospital, Chiayi 600, Taiwan; 5 Department of Psychiatry, Chiayi Branch, Taichung Veterans General Hospital, Chiayi 600, Taiwan

**Keywords:** epidemiology, hyperlipidemia, chronic obstructive pulmonary disease (COPD), risk factors

## Abstract

The coexistence of chronic obstructive pulmonary disease (COPD) and cardiovascular disease is common and causes poor prognoses. Hyperlipidemia is the most common risk factor for cardiovascular disease, but the association between hyperlipidemia and COPD remains ambiguous. This study aimed to investigate the risk of COPD development in patients with hyperlipidemia. This retrospective cohort study used information from the National Health Insurance Research Database in Taiwan. We enrolled 21,790 patients with hyperlipidemia and 87,160 control patients without hyperlipidemia for comparison, with a follow-up period of over 10 years. The incidence of new-onset COPD was higher in patients with hyperlipidemia (36.14 per 1000 person-years) than in the controls (22.29 per 1000 person-years). Patients with hyperlipidemia were 1.48 times more likely to develop subsequent COPD than the controls without hyperlipidemia (95% confidence interval 1.44 to 1.53, *p* < 0.001) following adjustments for age, sex, and comorbidities. In addition, nephropathy, hypertension, congestive heart failure, age, and sex (female) were potential risk factors for developing COPD in patients with hyperlipidemia. Patients with hyperlipidemia may have an increased risk of developing COPD.

## 1. Introduction

Hyperlipidemia is a common disorder characterized by elevated lipid levels in the human body. Hyperlipidemia is defined when a person has levels of low-density lipoprotein, total cholesterol, triglyceride (TG), or lipoprotein that are above the 90th percentile when compared with the general population, or when a person has a high-density lipoprotein (HDL) level below the 10th percentile in comparison with the general population [1]. 

Chronic obstructive pulmonary disease (COPD) is a prevalent disease characterized by persistent respiratory symptoms and restricted airflow [2]. The disease pathology involves abnormalities in the airway and/or alveoli and has a variety of causes, such as exposure to noxious particles or gases. Chronic inflammation leads to structural changes, including the narrowing of the small airways and destruction of the lung parenchyma.

COPD is one of the most serious public health issues in people aged 40 years and older [3]. The disease affects over five percent of the population worldwide and is associated with high mortality and morbidity [4,5]. COPD necessitates immense resource utilization due to its high prevalence and chronicity, which includes frequent office visits to clinicians, hospitalizations, and demand for persistent therapy [6].

Like other chronic diseases, COPD is associated with a plethora of comorbidities. COPD and cardiovascular disease frequently occur together and their coexistence is associated with worsened prognosis and higher mortality [7]. Hyperlipidemia is a major risk factor for cardiovascular diseases [2,8]. Numerous studies have evaluated the relationship between COPD and blood lipid profiles [9,10,11]. Some studies have shown that hyperlipidemia is more prevalent in patients with COPD [9,10], which indicates that hyperlipidemia may play a role in the pathophysiology of COPD.

However, the role of hyperlipidemia in COPD patients remains ambiguous. Most of those studies focused on hyperlipidemia in patients who had been diagnosed with COPD already. To the best of our knowledge, no previous study has examined whether hyperlipidemia affects the incidence of subsequent COPD, and there have been no large-scale investigations into the subject. The results may change our attitude toward COPD in patients with hyperlipidemia and particularly the pathophysiology between COPD and hyperlipidemia. Based on the above reasons, we hypothesized that hyperlipidemia is associated with the development of subsequent COPD, and we conducted a nationwide population-based study to investigate whether patients with hyperlipidemia have a higher risk of developing COPD. 

## 2. Materials and Methods

In 1995, the National Health Insurance (NHI) was established to cover nearly all Taiwanese residents [11]. The program is mandatory and provides all Taiwanese residents with comprehensive medical coverage, including outpatient care, inpatient services, emergency visits, and Chinese medicine. The National Health Insurance Research Database (NHIRD) contains complete clinical information, including prescription details and diagnostic codes using the format from the International Classification of Diseases, Ninth Revision, Clinical Modification (ICD-9-CM). The National Health Research Institutes (NHRIs) manage the NHIRD and data confidentiality is upheld by NHI Bureau guidelines. For our ongoing study, we used data from the Longitudinal Health Insurance Database 2000 (LHID 2000), an NHIRD dataset. The LHID 2000 contains the original claim data from 2,000,000 beneficiaries who were randomly selected from the NHIRD’s Registry for Beneficiaries in 2000. In comparing the patients in the LHID 2000 and those in the original NHIRD, the NHRIs of Taiwan confirmed that there were no significant differences in the sex distribution, age distribution, or average insured payroll-related amount.

We used information from the LHID 2000 to identify patients over the age of 20 who were diagnosed with hyperlipidemia for the first time between 1 January 2000, and 31 December 2004. Hyperlipidemia was designated as ICD-9-CM code 272, which is most common in current Taiwan NHIRD studies on hyperlipidemia [12,13]. We only selected patients who had at least two consensus diagnoses of hyperlipidemia during the observation period to ensure diagnostic validity. COPD was designated as ICD-9-CM codes 491, 492, and 496, rather than 490–496 for more precise correlation. Patients who had been diagnosed with COPD before enrollment were ruled out. Furthermore, we randomly chose the data of four control patients from the LHID 2000 for each hyperlipidemia patient included in our final cohort. The control patients were not diagnosed with hyperlipidemia during the observational period, and they were matched for age, sex, and enrollment date with each of the hyperlipidemia patients.

The cohort, which included patients with and without hyperlipidemia, was followed until COPD developed, death occurred, or the NHI system was terminated on 31 December 2013. The major clinical outcome in our study was COPD, as identified by primary physicians. We did not set a specific time interval between the two diseases as there is not enough evidence about the temporal effect of hyperlipidemia on COPD yet. In total, we identified 21,790 patients with hyperlipidemia. We randomly chose 87,160 patients without a history of hyperlipidemia to form a comparison cohort.

During the observational period, the incidence of newly diagnosed COPD in patients with hyperlipidemia and in controls was estimated and stratified by sex and age (<65 years or ≥65 years). An independent *t*-test was utilized to make comparisons between continuous variables. Chi-squared analysis was used to investigate the relationship of two categorical factors between the hyperlipidemia group and the control cohort. The crude risk factors were calculated by comparing the incidence rates between the hyperlipidemia and control groups in total and the specific stratification by age, sex, and follow-up period. We utilized a Cox proportional hazard model to seek possible influences of the confounding variables and to investigate whether hyperlipidemia raises the risk of COPD. The confounding variables included age, sex, and common comorbidities, such as diabetes (ICD-9-CM code 250), hypertension (ICD-9-CM codes 401–405), nephropathy (ICD-9-CM codes 580–589), depression (ICD-9-CM codes 296.2, 296.3, 300.4, and 311), cirrhosis (ICD-9-CM codes 571.5 and 571.6), autoimmune disease (ICD-9-CM codes 245.2, 250.01, 340, 358, 555.9, 556.9, 579, 696.0, 696.1, 710.1, and 710.2), congestive heart failure (ICD-9-CM code 428), alcoholism (ICD-9-CM codes 303, 571.0–571.3, 577.0), and obesity (ICD-9-CM code 278). Urbanization and monthly income levels were used to represent socioeconomic status. Urbanization was categorized into three groups: urban, suburban, and rural. The monthly income of patients, which was based on their insurance premium, was divided into no income, low income (monthly income < 20,000 New Taiwan Dollar (NTD)), median income (20,000 NTD ≤ monthly income < 40,000 NTD), and high income (monthly income ≥ 40,000 NTD). A Cox proportional-hazards regression model was used to identify variables that predicted COPD in the patients with hyperlipidemia. The variables that exhibited a fairly significant statistical correlation with COPD in the univariate analysis were entered using forward selection in multivariate analysis. Kaplan–Meier curves were used to examine the cumulative incidences of COPD between the hyperlipidemia patients and the control group. IBM SPSS Statistics (version 25.0 for Windows; IBM Corp., New York, NY, USA) was used to perform all statistical analyses. *p* < 0.05 was used to denote statistical significance.

## 3. Results

In total, 21,790 patients with hyperlipidemia and 87,160 control patients were eligible for the study. The demographic and clinical data for the two groups are shown in Table 1. The mean age of the patients was 42.54 years in each group (standard deviation of 13.26 both in the hyperlipidemia and control groups). There were 8.88 and 9.42 years of follow-up on average in the hyperlipidemia and control groups, respectively. The three-most-prevalent comorbidities in both groups were diabetes mellitus, hypertension, and nephropathy.

**Figure 1:**
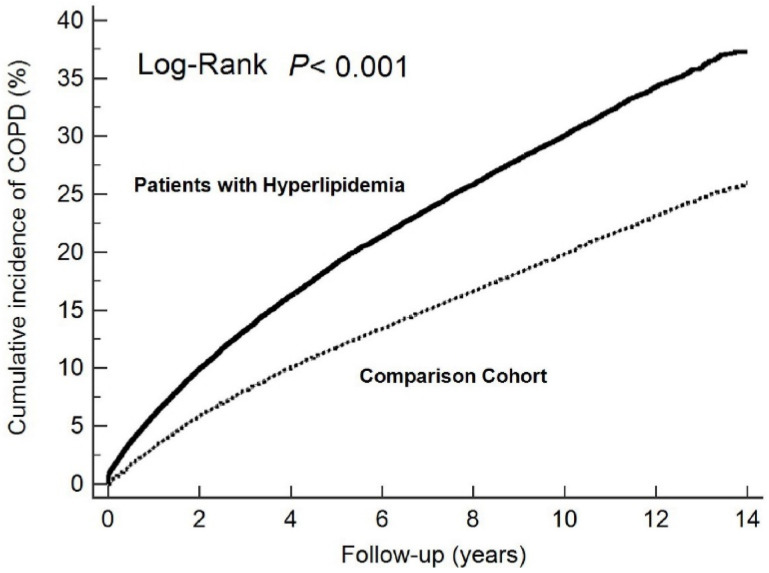
Cumulative incidence of newly diagnosed chronic obstructive pulmonary disease (COPD) in patients with (solid line) and without (dashed line) hyperlipidemia.

In total, 6995 patients were newly diagnosed with COPD in the hyperlipidemia group during the follow-up period. The patients in the hyperlipidemia cohort had a significantly greater incidence of developing COPD than those in the control cohort. Furthermore, a subanalysis of our study stratified by the number of years of follow-up revealed that the hyperlipidemia cohort had the highest risk ratio (RR) for developing COPD within one year of the hyperlipidemia diagnosis (Table 2). Even though the risk of developing COPD decreased over time, it remained statistically significant even more than ten years after the hyperlipidemia diagnosis. Patients with hyperlipidemia had a greater risk of developing COPD than patients in the control cohort (crude RR = 1.61, 95% confidence interval (CI) = 1.57–1.66), as indicated in Table 2. The cumulative incidence of newly diagnosed COPD in patients with and without hyperlipidemia is shown in Figure 1.

Cox proportional hazard regression analysis was used to determine the hazard ratio (HR) of newly diagnosed COPD in the hyperlipidemia patients and the control group. After adjusting for confounding factors, we detected the same relationship between hyperlipidemia and COPD (RR = 1.48, 95% CI = 1.44–1.53), as shown in Table 3.

In individuals with hyperlipidemia, we applied Cox proportional hazard regression analysis to identify risk factors for COPD. The results of the multivariate analysis revealed that nephropathy, hypertension, congestive heart failure, age, and sex (female) were risk factors for developing COPD in individuals with hyperlipidemia (Table 4).

## 4. Discussion

Our study of a large-scale, population-based retrospective cohort demonstrated several major findings. First, after adjusting for confounding factors, we found that patients with hyperlipidemia presented a 1.48-fold greater risk of subsequently developing COPD than our control cohort. Further analysis indicated that the risk factors for hyperlipidemia patients who subsequently developed COPD were nephropathy, hypertension, congestive heart failure, age, and sex (female).

The major advantage of this study is that it used nationwide population data to assess the risk of COPD in patients with hyperlipidemia. The strengths of using Taiwan’s NHIRD for medical research have been described [14], and they include the large sample size, long-term follow-up, and lack of selection and participation bias. Previous studies have demonstrated that patients with COPD have concurrent hyperlipidemia, particularly in terms of the TG and HDL levels [15,16]. To our knowledge, this is the first study to suggest that there is a temporal relationship between patients with hyperlipidemia and the development of new-onset COPD.

The vascular endothelium is a critical organ that dynamically regulates a variety of physiological and pathological processes. Endothelial cells serve as a barrier between the circulatory system and peripheral tissues, actively regulating vascular tone, blood flow, and platelet function [7]. The endothelium modulates vascular tone mainly through the synthesis of nitric oxide (NO) by the endothelial NO synthase isoform [17,18]. Endothelial dysfunction (ED) may result from the decreased synthesis of NO and increased degradation of NO due to interactions with superoxide anions [19]. ED is thought to be a systemic condition that affects not only the coronary arteries, but also the rest of the circulatory system [10]. 

Several reports have provided evidence that hyperlipidemia results in ED [19,20,21,22]. Many diverse pathways mediate ED caused by lipotoxicity, including increased oxidative stress and proinflammatory responses. In patients with obesity, metabolic syndrome, or diabetes, the effect of lipotoxicity on ED is amplified even more [23,24,25]. ED induced by hyperlipidemia may play an important role in increasing the incidence of subsequent COPD.

Recently, ED has been linked to pulmonary lesions in COPD, particularly emphysema [10]. Early research on the pathogenesis of COPD focused on the effects of injury on the extracellular matrix and pulmonary epithelial cells. In 1991, Dinh-Xuan and colleagues discovered that ED occurs in the pulmonary arteries of COPD patients with end-stage disease [26]. Peinado and colleagues indicated that ED is also an early feature of COPD [27]. Flow-mediated dilation assessed by ultrasound is commonly used to measure ED, and impairment in flow-mediated dilation directly correlates with the severity and prognosis of COPD [28,29,30,31]. Polverino and colleagues reviewed the evidence linking ED to COPD and identified at least seven possible pathways for the so-called vascular COPD phenotype [10].

Recent studies have found a clear link between chronic nephropathy and COPD, both of which contribute to ED [32,33]. One cohort study reported that COPD patients had significant persistent microalbuminuria, an indicator of widespread ED and renal injury, in comparison with controls [32]. COPD patients had greater endothelial cell apoptosis in small vessels in both the lungs and kidneys than smoker and non-smoker controls [33]. This evidence is compatible with the results of our analysis. 

Our multivariate analysis revealed many other variables that may be risk factors for developing COPD in hyperlipidemia patients. Previous studies have indicated that several variables contribute to the development of ED, including hypertension, congestive heart failure, age, and sex [34,35]. 

Recently, numerous studies have demonstrated that hypertension is a cause, rather than a result, of ED [36,37,38,39]. Several lines of evidence have suggested that ED deteriorates as blood pressure rises, and the degree of dysfunction is related to the magnitude of blood pressure elevation [40,41,42]. 

ED is related to congestive heart failure and its prognosis. Several studies showed a significant reduction in endothelium-mediated vasodilation in the peripheral arteries of patients with chronic heart failure [43,44,45,46]. Drexler and colleagues observed cytokines and decreased flow, indicating that reduced shear stress may play a role in the development of ED in heart failure [35].

A large number of studies have indicated that ED occurs during the human aging process, which may be attributed mostly to endothelial cell senescence [47,48]. Meyrelles and colleagues performed a study on old apolipoprotein E-deficient mice, which develop hyperlipidemia similar to humans, and showed that they had poorer endothelial function than younger mice. This result suggests that older hyperlipidemia patients may have more severe ED [49].

In women, endothelial function declines noticeably during the fifth decade of life, around the time of menopause. Furthermore, later in life, endothelial function declines at a substantially faster rate in women than in men [50,51]. Endothelial function deteriorates more rapidly in postmenopausal women due to the loss of the vasoprotective effects of sex hormones, particularly estradiol [52]. This decline in endothelial function may synergize with hyperlipidemia to induce worse ED in women than in men.

There are several limitations to this study. The first limitation is the lack of detailed information regarding tobacco use, environmental and occupational exposures, atopy, and genetic factors in the patient data collected from the NHIRD, because these factors can influence the risk of COPD development [53] and smoking plays an important role in COPD. Smoking is one of the most extensively studied lifestyle exposures concerning COPD [54,55,56]. An association between hyperlipidemia and smoking in the Chinese population has also been found [57]. The lack of related data for a potential confounder is a common limitation in the literature conducting secondary data analyses [58,59,60]. In this study, we could not directly account for smoking as a confounding variable from the NHIRD. Further research is still warranted about the effects of smoking on hyperlipidemia and subsequent COPD. Second, our study did not include any information about the severity and medical treatments for hyperlipidemia and COPD. Third, only patients requesting medical assistance were identified in the NHIRD Registry due to its claims-based study design. Thus, concerns about identification could cause the results to be overestimated or underestimated. Fourth, patients in the hyperlipidemia group generally had more comorbidities than the controls. Although we use statistical methods to eliminate biases, the differences among people who are interested in health or visit hospitals more frequently may result in more diagnoses of COPD.

Individualized data, such as smoking habits and medication for hyperlipidemia, could be collected in future studies. Additionally, another group in which patients with hyperlipidemia have COPD from the beginning could be set. This would help to investigate the characteristics and differences between patients with hyperlipidemia diagnosed with COPD after enrollment.

COPD is prevalent in developed countries and has a profound health burden. In our population-based retrospective study, we found that patients with hyperlipidemia had a statistically higher risk of subsequently developing COPD. Furthermore, in hyperlipidemia patients, we identified several potential risk factors for developing COPD, including nephropathy, hypertension, congestive heart failure, age, and sex (female). Future studies are needed to determine the underlying mechanisms of these associations. 

## Figures and Tables

**Table 1:** Baseline characteristics of patients with and without hyperlipidemia.

Demographic Data	Patients with Hyperlipidemia		Patients without Hyperlipidemia		*p* Value
	n	%	n	%	
Age, year *	42.54 (13.26)		42.54 (13.26)		
≥65	1571	8.0	7004	8.0	0.999
<65	20,039	92.0	80,156	92.0	
Sex					
Male	12,938	59.4	51,752	59.4	0.999
Female	8852	40.6	35,408	40.6	
Comorbidities					
Diabetes mellitus	3278	15.0	3471	4.0	<0.001
Hypertension	5536	25.4	7865	9.0	<0.001
Nephropathy	2052	9.4	4216	4.8	<0.001
Depression	297	1.4	653	0.7	<0.001
Cirrhosis	218	1.0	636	0.7	<0.001
Autoimmune disease	540	2.5	1112	1.3	<0.001
Congestive heart failure	266	1.2	516	0.6	<0.001
Alcoholism	490	2.2	945	1.1	<0.001
Obesity	292	1.3	164	0.2	<0.001
Degree of urbanization					<0.001
Urban	13,856	63.6	54,089	62.1	
Suburban	6621	30.4	27,736	31.8	
Rural	1313	6.0	5335	6.1	
Income					<0.001
High income	3667	16.8	12,028	13.8	
Medium income	4642	21.3	17,451	20.0	
Low income	10,009	45.9	43,761	50.2	
No income	3472	15.9	13,920	16.0	
Follow-up, years *	8.88 (4.00)		9.42 (3.74)		<0.001

* Mean (standard deviation).

**Table 2:** Incidence of chronic obstructive pulmonary disease in patients with and without hyperlipidemia.

	Patients with Hyperlipidemia		Patients without Hyperlipidemia		RR (95% CI) *	*p* Value
	No. of COPD	Incidence ^†^	No. of COPD	Incidence ^†^		
Total	6995	36.14	18,295	22.29	1.61 (1.57–1.66)	<0.001
Age, year						
≥65	900	81.98	2999	69.33	1.19 (1.10–1.28)	<0.001
<65	6095	33.38	15,296	19.67	1.69 (1.64–1.74)	<0.001
Sex						
Male	4021	35.12	10,607	21.96	1.59 (1.53–1.65)	<0.001
Female	2974	37.62	7688	22.75	1.64 (1.58–1.72)	<0.001
Follow-up, years						
0–1	1286	2276.50	2722	1344.67	1.72 (1.61–1.84)	<0.001
1–5	2829	310.19	7256	257.26	1.16 (1.11–1.21)	<0.001
5–10	2248	42.83	6381	30.67	1.45 (1.38–1.52)	<0.001
≥10	632	4.81	1936	3.32	1.46 (1.33–1.59)	<0.001

* Crude RR; ^†^ Indicates per 1000 person-years; COPD, chronic obstructive pulmonary disease; RR, risk ratio; CI, confidence interval.

**Table 3:** Analyses of risk factors for chronic obstructive pulmonary disease in patients with and without hyperlipidemia.

Predictive Variables	Univariate Analysis		Multivariate Analysis	
	HR (95% CI)	*p* Value	HR (95% CI)	*p* Value
Hyperlipidemia	1.61 (1.57–1.66)	<0.001	1.48 (1.44–1.53)	<0.001
Age (<65 = 0, ≥65 = 1)	3.09 (2.99–3.20)	<0.001	2.63 (2.52–2.73)	<0.001
Sex (male = 0, female = 1)	1.05 (1.02–1.07)	<0.001	1.05 (1.02–1.08)	<0.001
Comorbidities				
Diabetes mellitus	1.70 (1.63–1.78)	<0.001	1.08 (1.03–1.13)	0.001
Hypertension	2.07 (2.00–2.13)	<0.001	1.32 (1.28–1.37)	<0.001
Nephropathy	1.61 (1.54–1.69)	<0.001	1.25 (1.20–1.31)	<0.001
Depression	1.26 (1.11–1.42)	<0.001	1.15 (1.02–1.30)	0.026
Cirrhosis	1.71 (1.51–1.94)	<0.001	1.31 (1.15–1.49)	<0.001
Autoimmune disease	1.33 (1.21–1.45)	<0.001	1.15 (1.05–1.26)	0.004
Congestive heart failure	2.63 (2.36–2.94)	<0.001	1.29 (1.15–1.45)	<0.001
Alcoholism	1.35 (1.22–1.50)	<0.001	1.15 (1.03–1.27)	0.011
Obesity	1.49 (1.27–1.75)	<0.001	1.22 (1.04–1.44)	0.015
Degree of urbanization				
Urban	Reference		Reference	
Suburban	1.09 (1.06–1.12)	<0.001	1.06 (1.03–1.08)	<0.001
Rural	1.36 (1.30–1.43)	<0.001	1.24 (1.18–1.30)	<0.001
Income				
High income	Reference		Reference	
Medium income	1.04 (1.00–1.09)	0.075	1.05 (1.00–1.10)	0.033
Low income	1.16 (1.12–1.21)	<0.001	1.02 (0.98–1.06)	0.322
No income	1.26 (1.21–1.32)	<0.001	0.98 (0.94–1.03)	0.479

HR, hazard ratio; CI, confidence interval.

**Table 4:** Analyses of risk factors for chronic obstructive pulmonary disease in patients with hyperlipidemia.

Predictive Variables	Univariate Analysis		Multivariate Analysis	
	HR (95% CI)	*p* Value	HR (95% CI)	*p* Value
Age (<65 = 0, ≥65 = 1)	2.34 (2.18–2.51)	<0.001	2.07 (1.91–2.24)	<0.001
Sex (male = 0, female = 1)	1.07 (1.02–1.13)	0.004	1.07 (1.01–1.12)	0.011
Comorbidities				
Diabetes mellitus	1.18 (1.11–1.26)	<0.001	1.01 (0.95–1.08)	0.735
Hypertension	1.41 (1.34–1.48)	<0.001	1.19 (1.13–1.26)	<0.001
Nephropathy	1.33 (1.23–1.43)	<0.001	1.21 (1.12–1.30)	<0.001
Depression	1.13 (0.93–1.37)	0.226		
Cirrhosis	1.20 (0.95–1.51)	0.129		
Autoimmune disease	1.18 (1.03–1.37)	0.022	1.11 (0.96–1.28)	0.179
Congestive heart failure	1.94 (1.62–2.33)	<0.001	1.36 (1.13–1.63)	0.001
Alcoholism	1.09 (0.93–1.28)	0.280		
Obesity	1.15 (0.94–1.39)	0.171		
Degree of urbanization				
Urban	Reference		Reference	
Suburban	1.06 (1.01–1.12)	0.024	1.03 (0.98–1.09)	0.230
Rural	1.22 (1.11–1.34)	<0.001	1.14 (1.04–1.26)	0.006
Income				
High income	Reference		Reference	
Medium income	1.16 (1.07–1.25)	<0.001	1.15 (1.06–1.24)	0.001
Low income	1.33 (1.24–1.42)	<0.001	1.18 (1.10–1.27)	<0.001
No income	1.32 (1.21–1.43)	<0.001	1.06 (0.97–1.16)	0.191

HR, hazard ratio; CI, confidence interval.

## Data Availability

All data relevant to the study are available from the National Health Insurance Research Database (NHIRD), which is provided by the National Health Insurance (NHI) administration, Ministry of Health and Welfare of Taiwan, and the National Health Research Institutes (NHRIs) of Taiwan. It is not publicly available because only researchers or clinicians who have applied and signed an agreement with the NHRIs are eligible to apply for the National Health Insurance Research Database (NHIRD). The following is the official website of the NHIRD (https://nhird.nhri.org.tw/).

## Abbreviations

COPD, chronic obstructive pulmonary disease; TG, triglyceride; HDL, high-density lipoprotein; NHI, National Health Insurance; NHIRD, National Health Insurance Research Database; ICD-9-CM, International Classification of Diseases, Ninth Revision, Clinical Modification; NHRIs, National Health Research Institutes; LHID 2000, Longitudinal Health Insurance Database 2000; NTD, New Taiwan Dollar (NTD); RR, risk ratio; HR, hazard ratio; NO, nitric oxide; ED, endothelial dysfunction, BNHI, Bureau of National Health Insurance.
